# Supplementary material for: The Effect of Microbiome-Modulating Agents (MMAs) on Type 1 Diabetes: A Systematic Review and Meta-Analysis of Randomized Controlled Trials
Source: Nutrients. 2024 May 29;16(11):1675. doi: 10.3390/nu16111675 (PMC11174426; doi:10.3390/nu16111675)
Supplement: Supplementary file 1 [file nutrients-16-01675-s001.zip › nutrients-3014592-supplementary/Supplementary S2.pdf]

Supplementary S2 Details of searching strategy and screening process

ESM Table 1. Search strategy details

Databases	Search terms
**PubMed (n=561)**	("Diabetes Mellitus, Type 1"[Majr]) AND ("Probiotics"[Majr] OR "Prebiotics"[Majr] OR"inulin"[Majr]) OR "bifidobacterium"[Majr] OR "lactoccocus"[Majr] OR "butyrate"[Majr])； filter clinical trial
**ISI Web of Science (n=6)**	((TI=(type 1 diabetes) OR TI=(T1D) OR TI=(type-1 diabetes)) AND AB=(trial)) AND (TI=(probiotic) OR TI=(prebiotic) OR TI=(synbiotic) OR TI=(butyrate))
**Cochrane library (n=36)**	("Probiotics or Prebiotic or inulin or postbiotic or butyrate or synbiotic" AND "Type 1 diabetes Mellitus [MESH]" AND "#1 And #2")<br>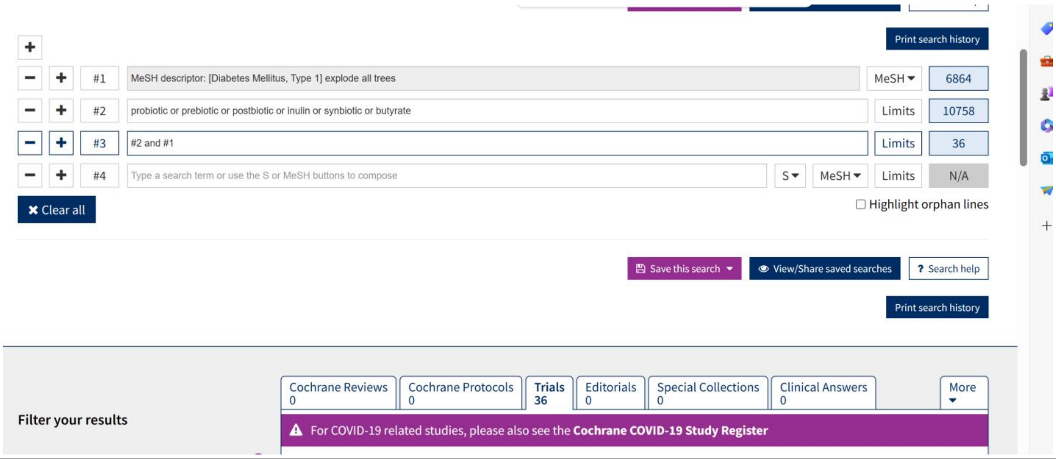
**Embase (n=91)**	probiotic:ti,ab,kw AND 'type 1 diabetes':ti,ab,kw
**CNKI (n=5)**	关键词（1 型糖尿病）精确 AND 关键词（益生菌）模糊；同义词扩展<br>Keywords (Type 1 diabetes in exact) AND Keywords (probiotic in fuzzy)
**Wangfang (n=4)**	(关键词=1 型糖尿病) AND 关键词=益生菌；资源类型=(中文期刊 OR 外文期刊 OR 学位论文 OR 会议论文)

**VIP**<br>**(n=128)**	题名或关键词（1 型糖尿病+insulin-depen- dent diabetes mellitus+type 1 diabetes+type 1 diabetes mellitus+type 1 diabetic+胰岛素依赖型糖尿病+一型糖尿病）AND 题名或关键词（益生菌+probiotics+微生态制剂+活菌制剂+益生菌剂+益生素+有益菌+有益微生物）

References of articles with full-text screened but excluded due to ineligibility

1. Savilahti E, Härkönen T, Savilahti EM, Kukkonen K, Kuitunen M, Knip M. Probiotic intervention in infancy is not associated with development of beta cell autoimmunity and type 1 diabetes. Diabetologia. 2018;61(12):2668-2670. doi:10.1007/s00125-018-4738-4

Excluded because participants are not diagnosed with T1DM.

2. Cabrera SM, Coren AT, Pant T, et al. Probiotic normalization of systemic inflammation in siblings of type 1 diabetes patients: an open-label pilot study. Sci Rep. 2022;12(1):3306. Published 2022 Feb 28. doi:10.1038/s41598-022-07203-6

Excluded because participants are not diagnosed with T1DM.

3. Mondanelli G, Orecchini E, Volpi C, et al. Effect of Probiotic Administration on Serum Tryptophan Metabolites in Pediatric Type 1 Diabetes Patients. Int J Tryptophan Res. 2020;13:1178646920956646. Published 2020 Sep 29. doi:10.1177/1178646920956646

Excluded because none of the published outcomes meets the outcomes needed for this review. The author did not reply the email.

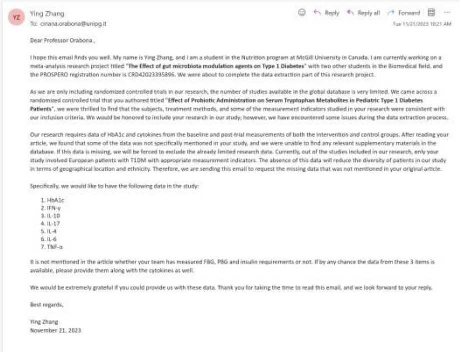

YZ Ying Zhang
To: ciriana.orabona@unipg.it
Reply Reply all Forward
Tue 11/21/2023 10:21 AM

Dear Professor Orabona ,

I hope this email finds you well. My name is Ying Zhang, and I am a student in the Nutrition program at McGill University in Canada. I am currently working on a meta-analysis research project titled "**The Effect of gut microbiota modulation agents on Type 1 Diabetes**" with two other students in the Biomedical field, and the PROSPERO registration number is CRD42023395896. We were about to complete the data extraction part of this research project.

As we are only including randomized controlled trials in our research, the number of studies available in the global database is very limited. We came across a randomized controlled trial that you authored titled "**Effect of Probiotic Administration on Serum Tryptophan Metabolites in Pediatric Type 1 Diabetes Patients**", we were thrilled to find that the subjects, treatment methods, and some of the measurement indicators studied in your research were consistent with our inclusion criteria. We would be honored to include your research in our study; however, we have encountered some issues during the data extraction process.

Our research requires data of HbA1c and cytokines from the baseline and post-trial measurements of both the intervention and control groups. After reading your article, we found that some of the data was not specifically mentioned in your study, and we were unable to find any relevant supplementary materials in the database. If this data is missing, we will be forced to exclude the already limited research data. Currently, out of the studies included in our research, only your study involved European patients with T1DM with appropriate measurement indicators. The absence of this data will reduce the diversity of patients in our study in terms of geographical location and ethnicity. Therefore, we are sending this email to request the missing data that was not mentioned in your original article.

Specifically, we would like to have the following data in the study:

1. HbA1c
2. IFN-γ
3. IL-10
4. IL-17
5. IL-4
6. IL-6
7. TNF-α

It is not mentioned in the article whether your team has measured FBG, PBG and insulin requirements or not. If by any chance the data from these 3 items is available, please provide them along with the cytokines as well.

We would be extremely grateful if you could provide us with these data. Thank you for taking the time to read this email, and we look forward to your reply.

Best regards,

Ying Zhang
November 21, 2023

4. Ross P. Expression of concern: Metabolic and genetic response to probiotics supplementation in patients with diabetic nephropathy: a randomized, double-blind, placebo-controlled trial. Food Funct. 2022;13(7):4229. Published 2022 Apr 4. doi:10.1039/d2fo90024f

Excluded because the majority of participants investigated on T2DM and there is a severe lack of data.

5. Soleimani A, Motamedzadeh A, Zarrati Mojarrad M, et al. The Effects of Synbiotic Supplementation on Metabolic Status in Diabetic Patients Undergoing

Hemodialysis: a Randomized, Double-Blinded, Placebo-Controlled Trial. Probiotics Antimicrob Proteins. 2019;11(4):1248-1256. doi:10.1007/s12602-018-9499-3
Excluded because participants are not diagnosed with T1DM.

6. Soleimani A, Zarrati Mojarrad M, Bahmani F, et al. Probiotic supplementation in diabetic hemodialysis patients has beneficial metabolic effects. Kidney Int. 2017;91(2):435-442. doi:10.1016/j.kint.2016.09.040
Excluded because participants are not diagnosed with T1DM.

7. Bell KJ, Saad S, Tillett BJ, et al. Metabolite-based dietary supplementation in human type 1 diabetes is associated with microbiota and immune modulation. Microbiome. 2022;10(1):9. Published 2022 Jan 19. doi:10.1186/s40168-021-01193-9
Excluded because the trial is not RCT.

8. Lai S, Lingström P, Cagetti MG, et al. Effect of Lactobacillus brevis CD2 containing lozenges and plaque pH and cariogenic bacteria in diabetic children: a randomised clinical trial. Clin Oral Investig. 2021;25(1):115-123. doi:10.1007/s00784-020-03342-0
Excluded because there is a lack of data, and the author did not reply.

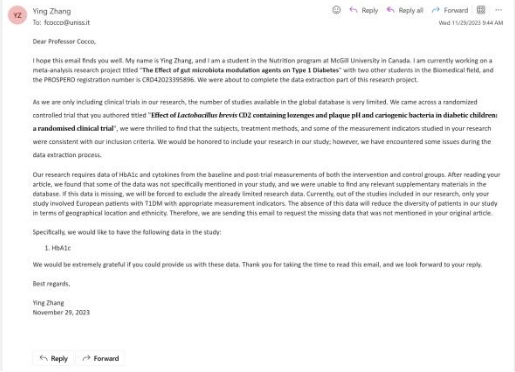
YZ Ying Zhang
To: fcocco@uniss.it
Reply Reply all Forward
Wed 11/29/2023 9:44 AM

Dear Professor Cocco,

I hope this email finds you well. My name is Ying Zhang, and I am a student in the Nutrition program at McGill University in Canada. I am currently working on a meta-analysis research project titled "**The Effect of gut microbiota modulation agents on Type 1 Diabetes**" with two other students in the Biomedical field, and the PROSPERO registration number is CRD42023395896. We were about to complete the data extraction part of this research project.

As we are only including clinical trials in our research, the number of studies available in the global database is very limited. We came across a randomized controlled trial that you authored titled "**Effect of *Lactobacillus brevis* CD2 containing lozenges and plaque pH and cariogenic bacteria in diabetic children: a randomised clinical trial**", we were thrilled to find that the subjects, treatment methods, and some of the measurement indicators studied in your research were consistent with our inclusion criteria. We would be honored to include your research in our study; however, we have encountered some issues during the data extraction process.

Our research requires data of HbA1c and cytokines from the baseline and post-trial measurements of both the intervention and control groups. After reading your article, we found that some of the data was not specifically mentioned in your study, and we were unable to find any relevant supplementary materials in the database. If this data is missing, we will be forced to exclude the already limited research data. Currently, out of the studies included in our research, only your study involved European patients with T1DM with appropriate measurement indicators. The absence of this data will reduce the diversity of patients in our study in terms of geographical location and ethnicity. Therefore, we are sending this email to request the missing data that was not mentioned in your original article.

Specifically, we would like to have the following data in the study:

1. HbA1c

We would be extremely grateful if you could provide us with these data. Thank you for taking the time to read this email, and we look forward to your reply.

Best regards,

Ying Zhang
November 29, 2023

Reply Forward

9. 牛文忠,丁显春．双歧杆菌乳杆菌三联活菌片对 1 型糖尿病肠道菌群和血清 IFN-γ、IL-4 的影响[J]．河南科技大学学报（医学版）,2016,36(3):196-198. DOI:10.15926/j.cnki.issn1672-688x.2016.03.011.
Excluded because the trial is not RCT.
