# Supplementary figures and images for: The Effect of Microbiome-Modulating Agents (MMAs) on Type 1 Diabetes: A Systematic Review and Meta-Analysis of Randomized Controlled Trials
Source: Nutrients. 2024 May 29;16(11):1675. doi: 10.3390/nu16111675 (PMC11174426; doi:10.3390/nu16111675)

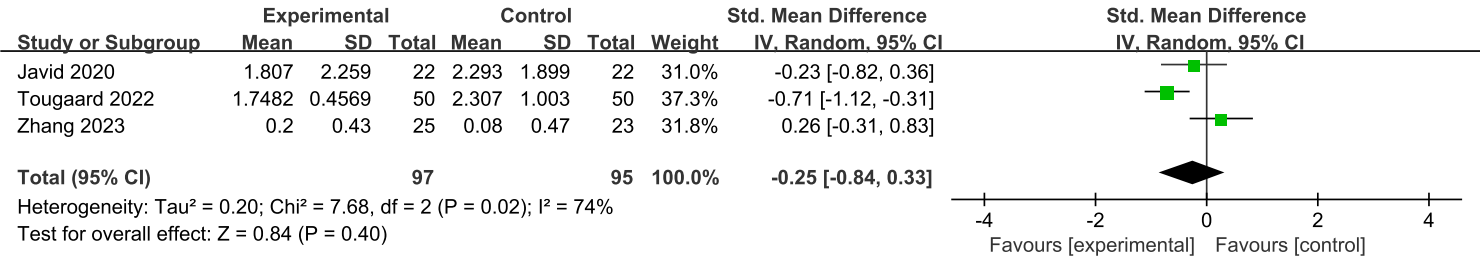

Supplement: Supplementary file 1 [file nutrients-16-01675-s001.zip › nutrients-3014592-supplementary/Supplementary S3/CRP.pdf]

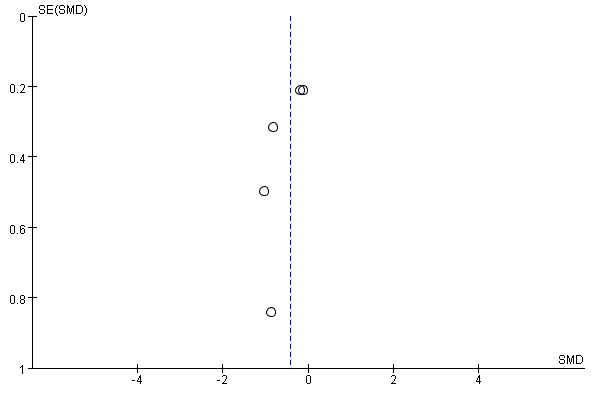

Supplement: Supplementary file 1 [file nutrients-16-01675-s001.zip › nutrients-3014592-supplementary/Supplementary S3/DIUFunnel plot.png]

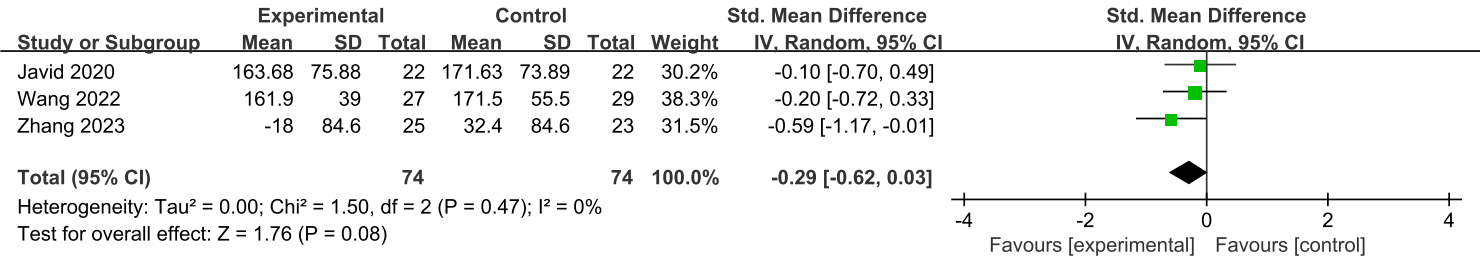

Supplement: Supplementary file 1 [file nutrients-16-01675-s001.zip › nutrients-3014592-supplementary/Supplementary S3/FBG.pdf]

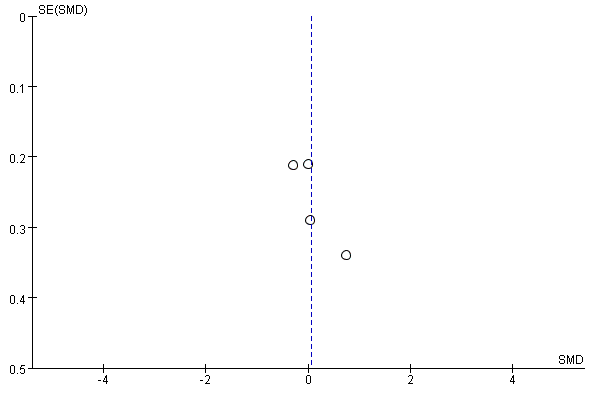

Supplement: Supplementary file 1 [file nutrients-16-01675-s001.zip › nutrients-3014592-supplementary/Supplementary S3/FCPFunnel plot.png]

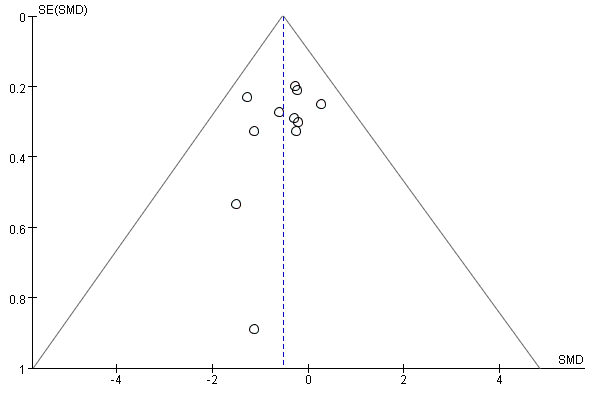

Supplement: Supplementary file 1 [file nutrients-16-01675-s001.zip › nutrients-3014592-supplementary/Supplementary S3/HbA1c Funnel plot.tif]

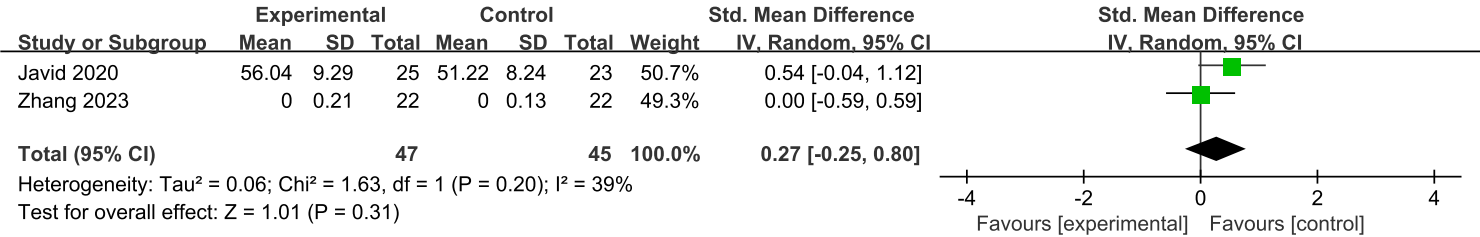

Supplement: Supplementary file 1 [file nutrients-16-01675-s001.zip › nutrients-3014592-supplementary/Supplementary S3/HDL.pdf]

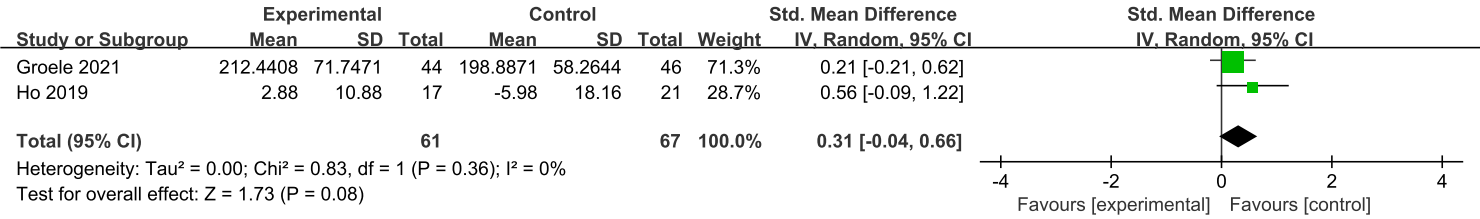

Supplement: Supplementary file 1 [file nutrients-16-01675-s001.zip › nutrients-3014592-supplementary/Supplementary S3/IL-10.pdf]

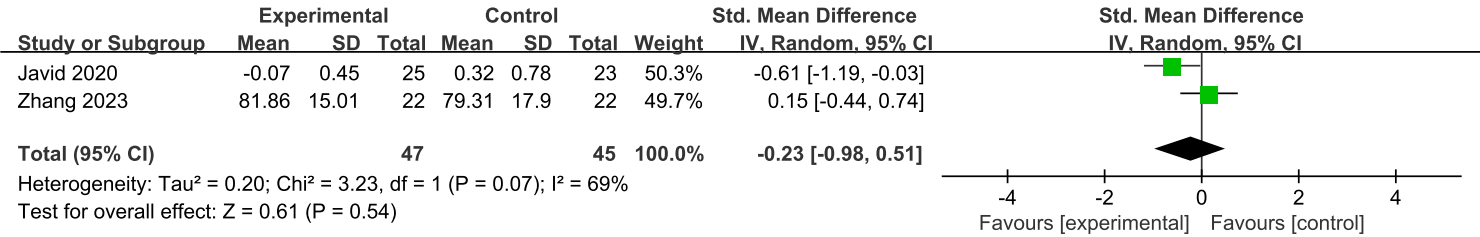

Supplement: Supplementary file 1 [file nutrients-16-01675-s001.zip › nutrients-3014592-supplementary/Supplementary S3/LDL.pdf]

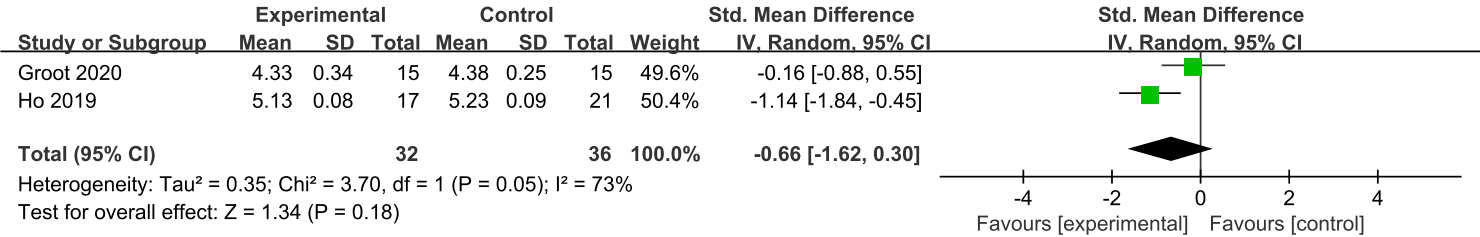

Supplement: Supplementary file 1 [file nutrients-16-01675-s001.zip › nutrients-3014592-supplementary/Supplementary S3/Shannon index.pdf]

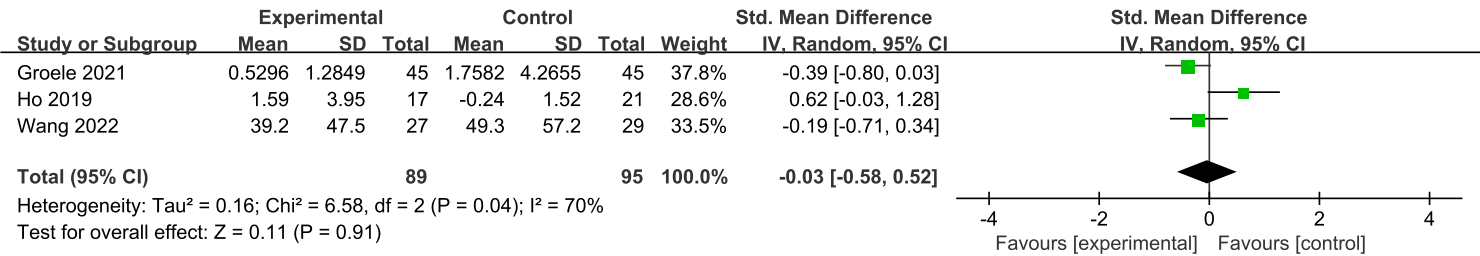

Supplement: Supplementary file 1 [file nutrients-16-01675-s001.zip › nutrients-3014592-supplementary/Supplementary S3/TNF-a.pdf]

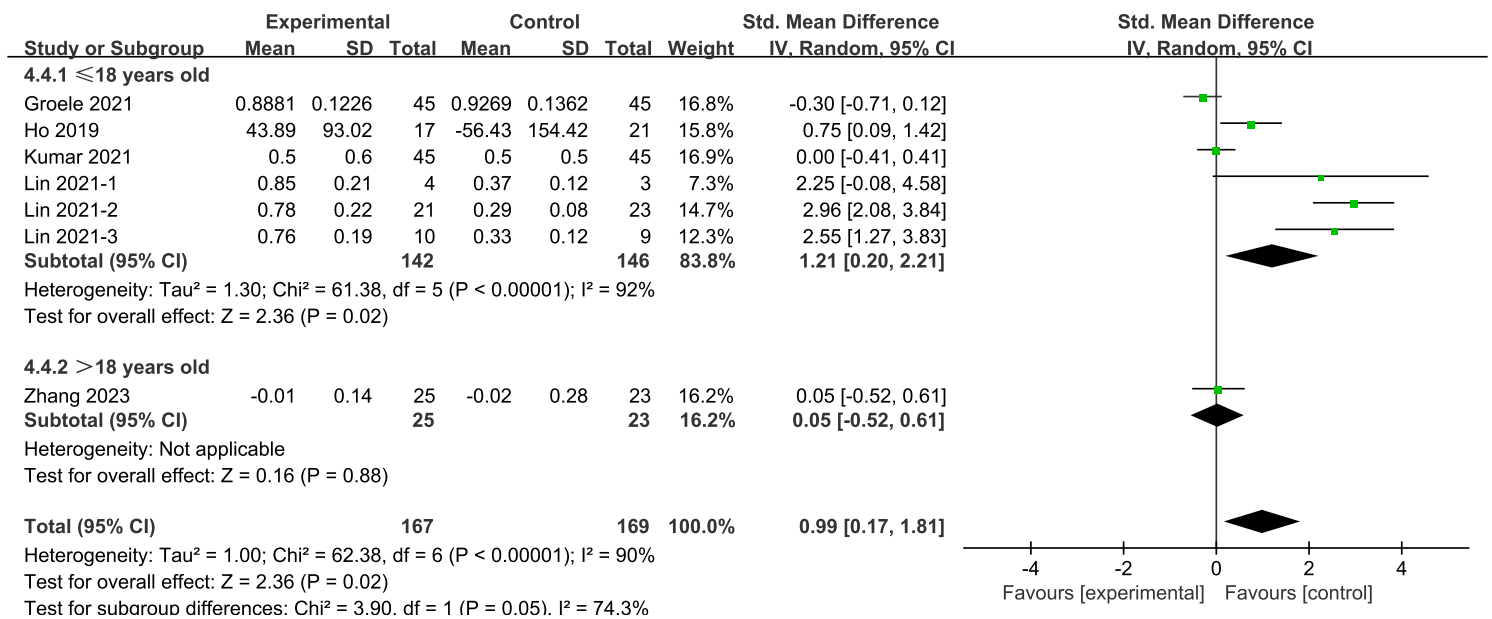

Supplement: Supplementary file 1 [file nutrients-16-01675-s001.zip › nutrients-3014592-supplementary/Supplementary S4/Age.pdf]

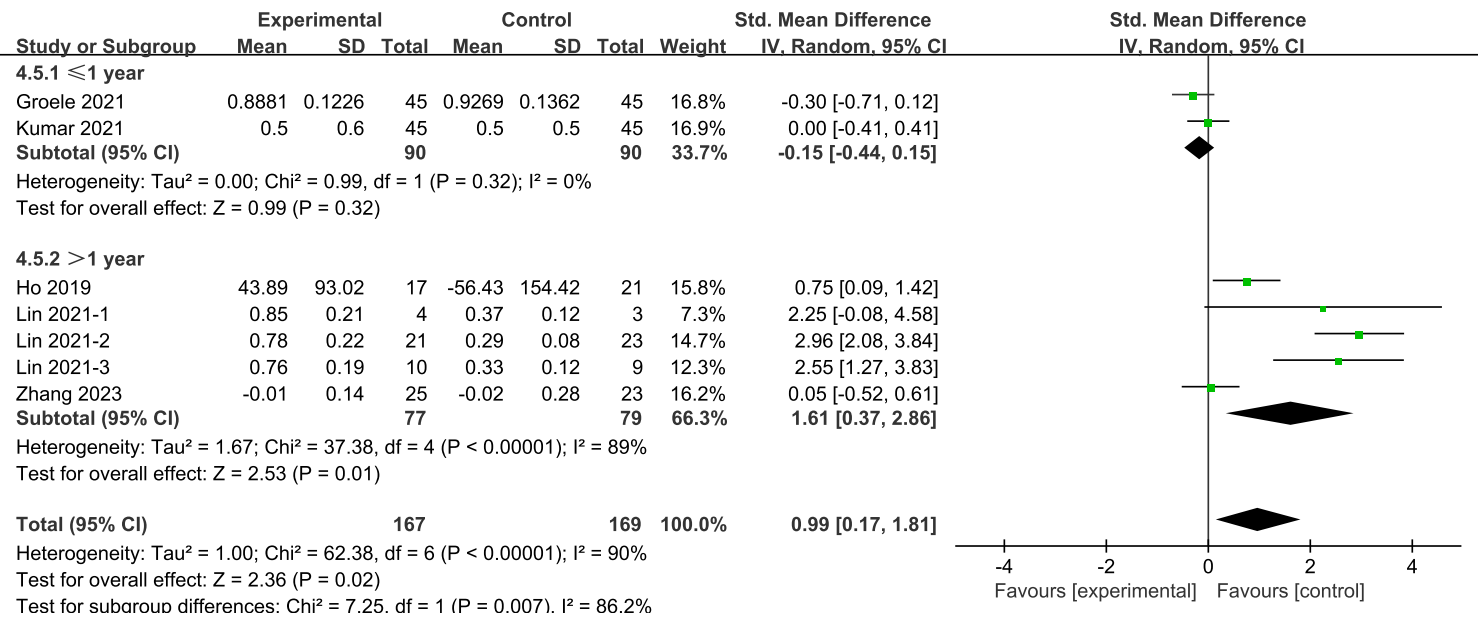

Supplement: Supplementary file 1 [file nutrients-16-01675-s001.zip › nutrients-3014592-supplementary/Supplementary S4/Disease duration.pdf]

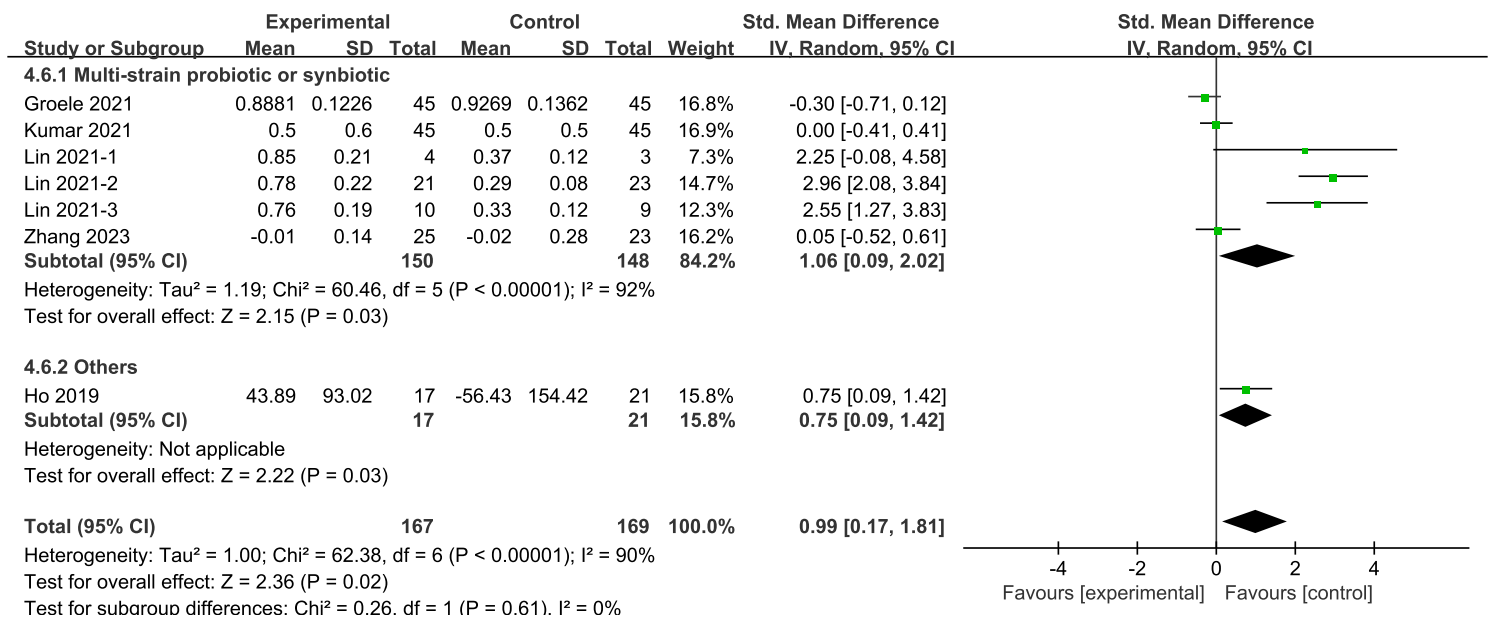

Supplement: Supplementary file 1 [file nutrients-16-01675-s001.zip › nutrients-3014592-supplementary/Supplementary S4/MMA.pdf]
